# Comparison of Clinico-Demographic and Histological Parameters Between Young and Old Patients With Oral Squamous Cell Carcinoma

**DOI:** 10.7759/cureus.48137

**Published:** 2023-11-01

**Authors:** Reshma Poothakulath Krishnan, Deepak Pandiar, Pratibha Ramani, Selvaraj Jayaraman, Raghunandhakumar Subramanian

**Affiliations:** 1 Oral Pathology and Microbiology, Saveetha Dental College and Hospitals, Saveetha Institute of Medical and Technical Sciences, Saveetha University, Chennai, IND; 2 Biochemistry, Saveetha Dental College and Hospitals, Saveetha Institute of Medical and Technical Sciences, Saveetha University, Chennai, IND; 3 Pharmacology, Saveetha Dental College and Hospitals, Saveetha Institute of Medical and Technical Sciences, Saveetha University, Chennai, IND

**Keywords:** nuclear pleomorphism, tumor-stroma ratio, tumor budding, keratinization, oral squamous cell carcinoma

## Abstract

Introduction: Among the epithelial malignancies of the head and neck region, oral squamous cell carcinoma (OSCC) arising from the oral mucosa is the commonest type. OSCC is common in the older population; however, recent epidemiological data indicate an increase in the incidence in the younger age group. The present study was designed to compare the clinicopathological characteristics of OSCC between young and old South Indian patients.

Methods: All the histopathologically confirmed cases of OSCC were retrieved from the department archives. Patients aged more than 40 years were considered Group I, and patients aged less than or equal to 40 were considered Group II. Age, gender, laterality, site, degree of keratinization, nuclear pleomorphism, pattern of invasion, lymphoplasmacytic infiltration, grade, tumor budding (TB), and tumor stroma ratio (TSR) were assessed.

Results: Among 510 patients reported with OSCC, 442 were aged above 40 years, and 68 were aged 40 years or younger. Nuclear pleomorphism, TB, and stroma-rich ratio were statistically higher in younger OSCC patients (p=0.00).

Conclusion: The results of our study support the fact that OSCC in younger individuals is more aggressive. Targeting TB and tumor stroma could provide new strategies for the management of OSCC.

## Introduction

Oral squamous cell carcinoma (OSCC) is the most common malignancy of the head and neck region and constitutes approximately 90% of oral cancers [1,2]. According to GLOBCAN statistics, the incidence of oral cavity and lip cancers is 10.2 per 100,000 population with 377,713 newly reported cases in 2020 [3]. OSCC ranks eighth globally and third in India [4]. Tobacco chewing, alcohol consumption, chronic irritation, smoking, and human papillomavirus are various known risk factors for OSCC [5]. The increase in morbidity and mortality among these patients remains a serious problem worldwide. Tumor size, delay in diagnosis, recurrence, and regional and distant metastasis are the leading causes of death.

OSCC is commonly reported in males in their sixth to eighth decades of life [6]. Although this is common in the older population, recent global epidemiological studies indicate an increase in the incidence of OSCC in younger patients (approximately 5-16.5%, below 40 years); however, the cause of this increasing trend remains unclear [7]. Studies have reported that oral cancers in younger individuals are more aggressive and show poor patient prognosis and survival rates [7]. Disagreements exist in the literature on the prognosis and survival outcome of OSCC in younger patients. OSCCs in the younger population are usually not associated with known risk factors such as tobacco chewing, smoking, or alcohol consumption [4]. Furthermore, younger patients usually do not present with a long-term history of carcinogen exposure, suggesting that the pathobiology might be different from those of older patients. Only a few studies have attempted to analyze various histopathological characteristics between the younger and older age groups.

Microscopic analysis along with detailed clinical examination plays an important role in the treatment planning, management, and determination of the overall survival of the patient. Histopathological features such as degree of keratinization, nuclear pleomorphism, inflammatory response, pattern of invasion, and the more recently described features such as tumor stroma ratio (TSR) and tumor budding (TB) are known to determine the prognosis of the patient [8,9]. TB is reported in various tumors such as colorectal, gastric, lung adenocarcinomas, pancreatic, and oesophageal cancer, including head and neck cancer. Tumor buds at the invasive tumor front connote poor prognosis and decreased survival of patients with colorectal cancer [8]. TSR is important for assessing the tumor microenvironment and is reported to have prognostic significance in gastric, colorectal, ovarian, and breast cancer patients. Previous studies also showed that patients with stroma-rich tumors had poor prognostic outcomes in carcinomas [9]. The histopathological features of the tumor, its morphology, and invasive pattern provide valuable information about the nature of the tumor. The present study was designed to compare the clinicopathological characteristics of OSCC between young and old South Indian patients.

## Materials and methods

Study design

This is a retrospective, observational study conducted in the Department of Oral Pathology and Microbiology of a private institution in Tamil Nadu, India. Prior clearance from the Institutional Ethical Committee was sought. Histopathologically confirmed cases of OSCC reported from January 2018 to April 2023 were retrieved from the department records and institutional electronic database. Only conventional OSCC cases with detailed demographic and clinical details were included in the study. Patients diagnosed with other histopathological variants of OSCC and recurrence cases were excluded.

Controversies exist over what age should be considered as "young patients." In various studies, an upper age of 40 years was considered because of the biological differences. In the present study, patients aged more than 40 years were considered the old age group (Group I), and patients aged less than or equal to 40 were grouped as young patients (Group II).

Clinicopathological evaluation

**Figure 1:**
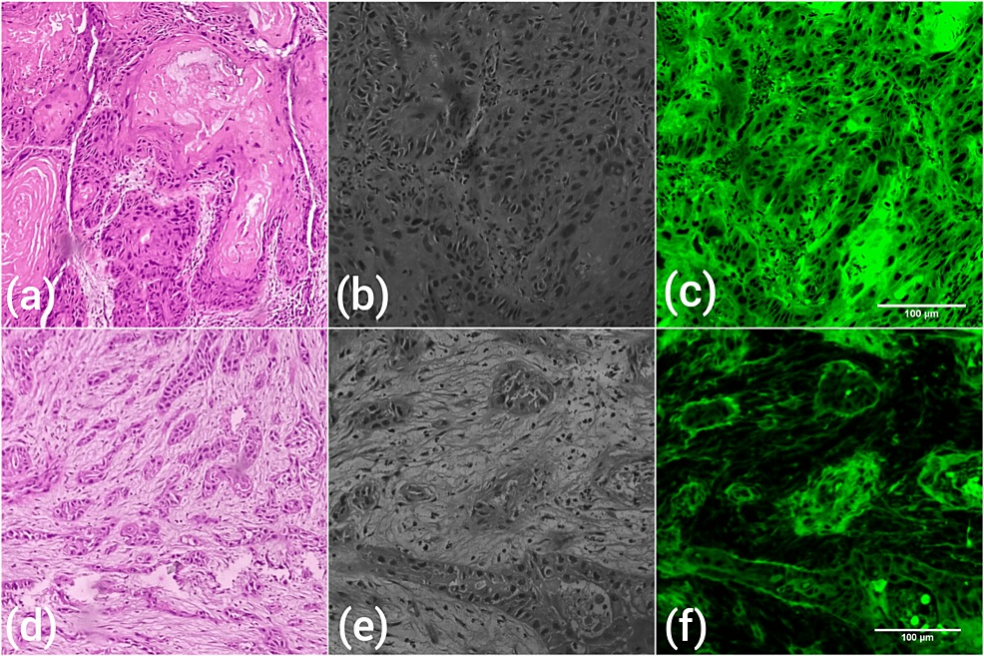
Photomicrographs showing stroma-poor oral squamous cell carcinoma (a-H&E, b-under fluorescent microscope without green filter, and c-under fluorescent microscope with green filter, 100X) and stroma-rich oral squamous cell carcinoma (d-H&E, e-under fluorescent microscope without green filter, and f-under fluorescent microscope with green filter, 100X). The basement membrane becomes clearly more evident under fluorescent microscopy.

Between Jan 2018 and April 2023, 510 patients were diagnosed with OSCC. Of these, 442 were aged more than 40 years, and 68 patients aged less than or equal to 40 years. Demographic and clinical details including age, gender, laterality, and site of lesion were noted. The slides were graded according to Bryne’s criteria as differentiated, moderately differentiated, and poorly differentiated independently by two oral pathologists (RPK & DP). If the staining was not satisfactory, archival blocks were retrieved, and H&E sections were made. Four parameters, namely, degree of keratinization, nuclear pleomorphism, pattern of invasion, and lymphoplasmacytic infiltration, were scored at the invasive front of the neoplasm on a four-point scale. The total score of 4-8 categorized the tumor as well-differentiated squamous cell carcinoma (WDSCC), 9-12 as moderately differentiated squamous cell carcinoma (MDSCC), and a score of more than 13 as poorly differentiated squamous cell carcinoma (PDSCC) [10]. Histopathological parameters such as TB and the TSR were also evaluated. TB is defined as the presence of less than five cancer cells (single/in groups) in the invasive front of the tumor [11]. The presence or absence of TB was noted for all included cases. TSR is the proportion of tumor cells relative to the surrounding stromal tissue. TSR was evaluated in the invasive front of the tumor as described by Sullivan et al. [12]. The area with the highest percentage of stroma and tumor islands located on four sides of the microscopic field were identified. The field with more than 50% stroma was classified as stroma-rich, and the field with less than 50% stroma was reported as stroma-poor [12]. The TSR was further validated using a fluorescent microscope with a green excitation filter to clearly distinguish the cancer cells and intervening stroma (Figure 1).

Statistical analysis

The above parameters were compared between the groups, and statistical analysis was conducted using Statistical Product and Service Solutions (SPSS, version 23) (IBM SPSS Statistics for Windows, Armonk, NY). Descriptive analysis, a Kappa test, and a chi-square test were used for comparison, and a p-value less than or equal to 0.05 was considered statistically significant.

## Results

Demographic data

Among the 510 patients who reported with OSCC, 442 were aged above 40 years, and 68 were aged 40 years or younger. The mean age of occurrence of OSCC in older patients was 55.80±9.7 and for younger patients was 36.43±3.32. Of the cases, 52.96% were reported on the left side of the oral cavity. Overall, the males outnumbered females with a ratio of 3.14:1 (M:F). Among the groups, the M:F ratio was 2.81:1 in Group I and 8.71:1 in Group II. Buccal mucosa and the lateral border of the tongue had the highest incidence of OSCC in both groups (Group I: 141 (31.9%), 120 (27.14%), Group II: 29 (42.64%), 23 (33.82%) respectively) (Table 1).

**Table 1:** Clinico-demographic details of oral squamous cell carcinoma patients

	Group I N (%)	Group II N (%)	P value
Gender			
Male	326 (73.75%)	61 (89.7%)	0.004
Female	116 (26.3%)	7 (10.29%)	
Laterality			
Right	209 (47.28%)	36 (52.94%)	0.385
Left	233 (52.71%)	32 (47.05%)	
Site			
Buccal mucosa	141 (31.90%)	29 (42.64%)	0.478
Lateral border of the tongue	120 (27.14%)	23 (33.82%)	
Others	181 (40.95%)	16 (23.52%)	

Degree of keratinization

There was no significant difference in the keratinization of cells in the invasive front between the age groups (p=0.244). The cells at the invasive front were highly keratinized in 151/442 (34.16%) and moderately keratinized in 183/442 (41.40%) of older age group patients. However, in the younger age group, 29/68 (42.64%) were highly or moderately keratinized. 100/442 (22.62%) in Group I and 10/68 (14.7%) in Group II showed minimal keratinization. Keratinization was not observed in 8/442 (1.8%) of older age group patients (Table 2).

**Table 2:** Histopathological characteristics of older and younger oral squamous cell carcinoma patients WDSCC, well-differentiated squamous cell carcinoma; MDSCC, moderately differentiated squamous cell carcinoma; PDSCC, poorly differentiated squamous cell carcinoma

	Group I N (%)	Group II N (%)	P value
Degree of keratinization			
Highly	151 (34.16%)	29 (42.64%)	0.244
Moderately	183 (41.4%)	29 (42.64%)	
Minimal	100 (22.62%)	10 (14.7%)	
No keratinization	8 (1.8%)	0	
Nuclear polymorphism			
Little	64 (14.47%)	3 (4.41%)	0.000
Moderately abundant	301 (68.09%)	25 (36.76%)	
Abundant	67 (15.15%)	25 (36.76%)	
Extreme	10 (2.26%)	15 (22.05%)	
Pattern of invasion			
Pushing, well-delineated infiltrating borders	90 (20.36%)	12 (17.64%)	0.036
Infiltrating- solid cords, bands, and or strands	172 (38.91%)	29 (42.64%)	
Small groups of cords of infiltrating cells	162 (36.65%)	19 (27.94%)	
Marked and widespread cellular dissociation in small groups of cells (n<15)	18 (4.07%)	8 (11.76%)	
Lymphoplasmacytic infiltrate			
Marked	114 (25.79%)	28 (41.17%)	0.061
Moderate	234 (52.94%)	29 (42.64%)	
Slight	90 (20.36%)	11 (16.17%)	
None	4 (0.9%)	0	
Histopathological diagnosis			
WDSCC	268 (60.63%)	41 (60.29%)	0.855
MDSCC	155 (35.06%)	25 (36.76%)	
PDSCC	19 (4.29%)	2 (2.94%)	
Tumor budding			
Present	125 (28.28%)	35 (51.47%)	0.000
Absent	317 (71.71%)	33 (48.52%)	
Tumor stroma ratio			
Stroma rich	57 (12.89%)	33 (48.52%)	0.000
Stroma poor	385 (87.10%)	35 (51.47%)	

Nuclear pleomorphism

The younger age group showed statistically higher nuclear pleomorphism than the older age group (p=0.00). Extreme nuclear pleomorphism was noted in 10/442 (2.26%) of Group I and 15/68 (22.05%) of Group II OSCC cases. Abundant pleomorphism was seen in 67/442 (15.15%) of older age group and 25/68 (36.76%) of younger patients (Table 2).

Pattern of invasion

There was no significant difference in invasive patterns between the age groups (p=0.036). Most of the patients in both age groups showed tumor cells infiltrating predominantly as solid cords, bands, or strands. Widespread cellular dissociation of the tumor cells was seen in 18/442 (4.07%) of Group I and 8/68 (11.76%) of Group II patients. Infiltration as tumor islands with broad well-delineated borders was noted in 90/442 (20.36%) of older and 12/68 (17.64%) of younger age groups (Table 2).

Lymphoplasmacytic infiltrate

No significant difference in the amount of lymphoplasmacytic infiltrate was noted between the two age groups (p=0.061). Of Groups I and II, 234/442 (52.94%) and 29/68 (42.64%) of patients, respectively, showed moderate lymphoplasmacytic infiltrate. Moreover, 90/442 (20.36%) of older patients and 11/68 (16.17%) of younger patients showed minimal inflammatory infiltrate (Table 2).

Histopathological grading

Based on the differentiation of the tumors, most of the cases were diagnosed as WDSCC in both groups. Additionally, 268/442 (60.63%) of the tumors in the older age group and 41/68 (60.29%) in the young age group were well differentiated; 155/442 (35.06%) in Group I and 25/68 (36.76%) in Group II were moderately differentiated; and 19/442 (4.29%) in Group I and 2/68 (2.94%) in Group II showed poor tumor grade (p=0.855) (Table 2).

TB

TB was noted significantly higher in the younger population when compared to the older population. Specifically, 35/68 (51.47%) of the younger patients showed tumor buds, while in the older age group, only 125/442 (28.28%) showed TB (p=0.00) (Table 2).

TSR

The high content of tumor stroma was noted in the younger population when compared to the older population. Stroma-rich tumor invasive front was noted in 57/442 (12.89%) of older age group, and 33/68 (48.52%) of younger age group patients. Stroma-poor invasive fronts were noted in 385/442 (87.10 %) of Group I and 35/68 (51.47%) of Group II patients (p=0.00) (Table 2).

## Discussion

HPV, chronic irritation, immunosuppression, syndromes (e.g., Bloom syndrome), hereditary disorders (e.g., dyskeratosis congenita), Fanconi anemia, and genetic predisposition are considered to play a major role in the etiopathogenesis of OSCC in the younger population [13]. It has also been stated that these tumors differ from the conventional OSCC in their clinical behavior and pathophysiology. The present study compared the clinicopathological characteristics of OSCC occurring in younger patients and older populations to highlight the clinico-demographic profile and histopathological characteristics in the Tamilian cohort.

It was noted that 13.33% of younger patients were diagnosed with OSCC between 2018 and 2023 in our institution. Other studies have reported 5.8-6.6% of cases in the younger age group [14]. This difference could be because of the different etiological factors, food habits, exposure to environmental carcinogens, and inherent genetic makeup of the younger patients in India. The use of tobacco products in various forms like bidi, gutka, zarda, cigarettes, mawa, khaini, and kharra is popular among the Indian population [15,16]. The above products significantly increase the risk of OSCC. Possibly, the use of these products from a younger age and their impaired ability to metabolize carcinogens because of lifestyle changes could be the reason for the increased incidence of oral cancer in the younger population of India.

In the present study, the mean age for Group I was found to be 55.80±9.7 and Group II to be 36.43±3.32. Similar results were reported from other parts of India. Abdulla et al. from Karnataka reported the mean age to be 39.62 years [4]. However, the Western population showed a marginally lower mean age when compared to the Indian scenario. Mneimneh et al. from the USA reported the mean age of the younger population as 34 years [13]. This difference could be attributed to the prevalence of disease-specific risk factors among the population. Generally, OSCC was noted more commonly in men than in women with male to female ratio of 2.81:1 in Group I and 8.71:1 in Group II. The M:F ratio was higher in younger patients than in the older population, suggesting a complex interplay between genetic sensitivity, environmental carcinogens, and lifestyle changes. Differences in exposure to carcinogens (smoking and alcohol consumption) and their easy availability could be other important contributing factors to the increased incidence of OSCC in younger males.

Buccal mucosa and the lateral border of the tongue were the most common sites for OSCC in younger patients. Mohideen et al. and Abdulla et al. from South India reported similar results [4,7]. The buccal mucosa is one of the common sites for OSCC in South India because of the placement of quid in the buccal/labial vestibule. Additionally, genetic predisposition also plays a major role. Young patients are mutagen-sensitive and show increased chromosomal breaks, low DNA repair capacity, and increased single nucleotide polymorphism [17]. Single nucleotide polymorphism of XPD and XRCC1 (at C26304T and G28152A) genes are reported as relatively high in younger head and neck squamous cell carcinoma (HNSCC) patients [18]. Furthermore, Foulkes et al. reported a 3.6-fold increased relative risk of head and neck carcinoma (HNC) in individuals with a family history of HNC-diagnosed first-degree relatives [19]. Combining these findings, possibly, the younger patients show an increased single nucleotide polymorphism in repair genes leading to increased vulnerability to the side effects of carcinogens causing chromosomal alternations and cancer. HPV infection, trauma, and oral microbiome through the production of carcinogenic factors are known to cause OSCC of the tongue in younger patients. PIK3CA, TP53, NOTCH1, FAT1, CDKN2A, CASP8, and MLL2 are commonly mutated genes in OSCCs [20]. A recent molecular study linked CDKN2A-HGF-MET and WNT-CTNNB1-STK11 pathways to the tongue OSCC in patients below 45 years [21].

Even though various histopathological parameters are used as prognosticators in OSCC, the significance of these has not been evaluated widely in the younger age group. We retrospectively evaluated the microscopic slides from the younger and older groups to determine any defining histopathological characteristics in young individuals. It was observed that higher nuclear pleomorphism, increased TB, and stroma-rich TSR ratio were evident in younger OSCC patients.

The nucleus controls the cell function and metabolism and reflects the biological potential of a cell. Nuclear analysis is well known to predict the biological nature of the tumor, its aggressiveness, and metastasis. Increased nuclear pleomorphism is associated with poor disease-free survival and increased clinical aggressiveness in various tumors like oropharyngeal squamous cell carcinoma, breast carcinoma, and OSCC [22]. High nuclear pleomorphism was also reported by Rahaman et al. in young OSCC patients [23]. The nucleus in young cases was more coarse or vesicular, anaplastic, and showed multiple prominent nucleoli. It is well known that the nuclear envelope controls the structure and function of the nucleus, connects the nucleoskeleton to the cytoskeleton, and plays an important role in signal transduction [24]. Nuclear abnormalities in cancerous cells disturb the cell cycle, influence cellular function, and lead to genomic instability. Fischer et al. reported that Lamin, a structural protein of nuclear membrane is disrupted in colorectal cancers causing abnormal signalling of PI3K-AKT-mTOR pathway, promoting cell motility [24]. Cell motility facilitates the dissemination and migration of cancer cells. Hence, the increased nuclear pleomorphism is supposedly related to the increased dysregulation of signaling pathways, altered gene expression patterns, and altered chromatin structure in younger individuals.

TB is the deployment of cancer cells from the bulk of the tumor. It correlates with adverse clinicopathological characteristics, increased lymph node metastases, distant metastases, decreased overall survival, and poor prognosis in colorectal cancers, breast cancers, and OSCCs [8]. In the present study, the younger age group showed increased TB and stroma-rich TSR. Dawson reported that the molecular background of the tumor plays a major role in the initiation of TB [25]. Tumor buds correlate with the epithelial-mesenchymal transition and appear more atypical than the main body of the tumor [25]. Grigore et al. suggested that the tumor buds show a partial epithelial-mesenchymal transition, with the expression of both epithelial and mesenchymal characteristics (hybrid E/M) [26]. Strong associations between TB and dysregulation of E cadherin and stromal alpha SMA-positive myofibroblasts have also been reported [27]. Tumor buds are known to express HIF-1a-mediated hypoxic tumor phenotype, degrade the underlying connective tissue stroma, and infiltrate the blood vessels/lymphatics [28]. A study performed by Mneimneh et al. demonstrated decreased survival and increased metastasis in younger individuals [13]. In the present study, 70% of younger patients who underwent surgical excision of the tumor showed nodal metastasis. Moreover, Wyk et al. reported that TB is associated with more stroma and decreased inflammatory infiltrate in colorectal cancers [28]. Cancers with a stroma-rich tumor indicate increased interaction between the stroma and tumor cells and increased release of stromal growth factors resulting in aggressive clinical behavior. Stroma-rich types are also known to further increase the depth of invasion, perineural invasion, and single-cell invasion [9]. These findings and our results further support the fact that the OSCCs in younger individuals are more aggressive.

Results of the present study suggest that the common type of OSCC is WDSCC irrespective of the age groups. The marked lymphoplasmacytic infiltrate was noted in 41.17% of younger OSCC patients and 25.79% of older patients. Pro-tumorigenic inflammatory response promotes cancer progression by shaping the tumor microenvironment (TME), blocking anti-tumor immunity, and activating tumor-promoting signals. Inflammation elevates the level of cytokines, nuclear factor kappa B, microRNAs, RONS, and prostaglandins that affect cancer proliferation, senescence, angiogenesis, DNA mutation, and methylation [29]. Although the inflammatory cells are considered to be a double-edged sword in cancer progression, further evaluation and characterization of the inflammatory cells in younger patients are required to understand the role of the tumor-associated inflammatory response, e.g., tumor-associated macrophages (TAM) in TME. Additionally, despite having higher lymphoplasmacytic infiltrate, the immune response might be altered in younger patients because of an imbalance between immune exhaustion and activation warranting further genetic analysis. Studies have reported that TP53 mutation, seen commonly within exons 5 to 9 in head and neck squamous cell carcinoma, occurs in a different location in younger patients [30]. Truncating-type TP53 mutation, which is usually associated with poor prognosis, is seen at a higher rate in younger individuals [30]. Several specific gene mutations are also reported in younger OSCC patients (e.g., INK4a, CDKN2A, SDHB, and RECQL4) [30].

Limitations

There are a few inherent limitations of the present study. The clinicopathological data was collected from the department records of a single institution with a comparatively lesser sample size and, hence, it may not be representative of the generalized Tamilian population. Furthermore, within the limitations of the study including the time, evaluation of confounding factors, and multivariate analysis could not be done.

## Conclusions

It can be concluded that increased nuclear pleomorphism, stroma-rich TSR, and increased TB were noted in younger OSCC cases. The results of our study further support the fact that OSCC in younger individuals is more aggressive. Targeting TB and tumor stroma could provide new strategies for the management of OSCC. However, further studies are required to unveil the molecular pathogenesis and pathophysiological mechanisms associated with OSCC in younger populations.
